# Management of early gastric cancer that meet the indication for radical lymph node dissection following endoscopic resection: a retrospective cohort analysis

**DOI:** 10.1186/s12893-017-0268-0

**Published:** 2017-06-20

**Authors:** Satoru Kikuchi, Shinji Kuroda, Masahiko Nishizaki, Tetsuya Kagawa, Hiromitsu Kanzaki, Yoshiro Kawahara, Shunsuke Kagawa, Takehiro Tanaka, Hiroyuki Okada, Toshiyoshi Fujiwara

**Affiliations:** 1 Department of Gastroenterological Surgery, Okayama University Graduate School of Medicine, Dentistry and Pharmaceutical Sciences, 2-5-1 Shikata-cho, Kita-ku, Okayama, 700-8558 Japan; 2 Department of Endoscopy, Okayama University Hospital, Okayama, 700-8558 Japan; 3 Department of Diagnostic Pathology, Okayama University Hospital, Okayama, 700-8558 Japan; 4 Department of Gastroenterology and Hepatology, Okayama University Graduate School of Medicine, Dentistry and Pharmaceutical Sciences, Okayama, 700-8558 Japan

**Keywords:** Early gastric cancer, Endoscopic resection, Lymph node metastasis

## Abstract

**Background:**

Endoscopic resection (ER) has been widely accepted as the standard treatment for early gastric cancer (EGC). However, in patients considered to have undergone non-curative ER due to their potential risk of lymph node metastasis (LNM), additional gastrectomy is recommended. The aim of the present study was to identify EGC patients after non-curative ER at high risk of LNM.

**Methods:**

A total of 150 patients who had undergone ER for EGC were diagnosed as non-curative ER due to their potential risk of LNM. Clinicopathological data and clinical outcomes were examined retrospectively.

**Results:**

Additional gastrectomy with lymph node dissection was performed in 73 patients, and the remaining 77 patients were followed-up without additional gastrectomy. In patients who underwent additional gastrectomy, 8 patients had local residual tumor, and 8 patients had LNM, which were limited in the peritumoral nodes. Only lymphatic invasion (*p* = 0.012) was a statistically significant factor for LNM. The 5-year overall survival and recurrence-free survival were not significantly different between patients with and without additional gastrectomy.

**Conclusion:**

Additional gastrectomy with lymph node dissection is recommended for patients who were diagnosed as non-curative ER with lymphatic invasion, and minimizing the extent of lymph node dissection may be allowed for these patients.

## Background

Gastric cancer is the world’s third leading cause of cancer mortality, responsible for 723,000 deaths each year [1]. With advances in diagnostic techniques and the increasing prevalence of screening programs, the percentage of early gastric cancer (EGC) cases is reaching nearly 60% in Japan [2, 3]. Endoscopic resection (ER) including endoscopic mucosal resection (EMR) and endoscopic submucosal dissection (ESD) has been widely accepted as the standard treatment for EGC patients when the risk of lymph node metastasis (LNM) is negligible [4, 5]. However, endoscopic diagnosis of EGC before ER is not always accurate, and some patients are diagnosed as non-curative ER due to the potential risk of LNM histologically after ER [5–7]. In patients diagnosed as non-curative ER, additional gastrectomy with lymph node dissection is generally recommended [7]. However, the LNM rate of patients who have undergone additional gastrectomy after non-curative ER is less than 10% [8–10]. The aim of the present study was to investigate the optimal treatment strategies for non-curative ER patients with a potential risk of LNM based on retrospective analysis in a single institution.

## Methods

### Patients

From January 2004 to August 2013, 707 patients with EGCs treated with ER, including EMR and ESD, at the Endoscopy Center of Okayama University Hospital, Okayama, Japan, were retrospectively studied. A total of 182 patients (25.7%) were subsequently diagnosed as non-curative ER after histological evaluation based on the Japanese gastric cancer treatment guidelines 2010 (version 3) [7] and they were classified into those with a positive hiatal margin (HM) only (*n* = 32), and those with a potential risk of LNM (*n* = 150). The clinical records of 150 patients after non-curative ER due to their potential risk of LNM were analyzed retrospectively with regard to clinicopathological findings of ER specimens, additional gastrectomy after ER, histology of surgical specimens, and prognosis.

The formalin-fixed specimens resected by ER were examined histologically using serial sections 2 mm in width according to the Japanese Classification of Gastric Carcinoma [11]. Lymphatic or venous infiltration was evaluated by examination of hematoxylin and eosin (H&E) stained sections. Curability was evaluated based on the histological criteria for curative ER [7]. Non-curative ER was defined as potential risk of LNM or positive lateral resection margin.

For all patients with a potential risk of LNM, additional gastrectomy with lymph node dissection was recommended, but for some patients, strict follow-up was selected due to the surgical risk, other primary cancers and new disorders after gastrectomy. They were divided into two subgroups: patients who underwent additional gastrectomy with lymph node dissection and those who received strict follow-up without gastrectomy.

Surgical specimens were examined according to the recommendations of the Japanese Classification of Gastric Carcinoma [11]. The entire resected stomach area was divided into 5-mm-wide slices, and LNMs were evaluated in the central portion of each lymph node. Local residual tumor was defined as any cancer diagnosed histologically at the ER site.

### Statistical analysis

Univariate analysis was performed using Fisher’s exact test or the χ^2^ test. Variables showing a univariate association (*p* < 0.50) were also subjected to multivariate analysis. Multivariate analysis was performed using logistic regression analysis to identify independent predictors related to LNM and local residual tumor. *P* values <0.05 were considered statistically significant. Clinical outcomes of patients who had additional gastrectomy and those who underwent strict follow-up were collected and analyzed in April 2017. Overall survival (OS) and recurrence-free survival curves were calculated by the Kaplan-Meier method. Statistical analysis was performed using JMP 11.2 (SAS Institute, Cary, NC, USA).

## Results

**Fig. 1:**
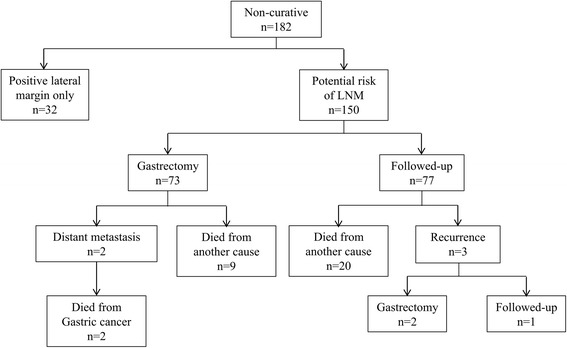
Clinical course of patients with non-curative endoscopic resection. LNM, lymph nodes metastasis

Seventy-three patients (49.7%) underwent additional gastrectomy **(**Fig. 1 **)**. The remaining 77 patients did not undergo additional gastrectomy by the reason of patient choice, high surgical risk, and other concomitant cancer. The patient’s clinical courses are summarized in Fig. 1. The demographics and clinical background characteristics of the 73 patients who underwent additional gastrectomy and the 77 who underwent strict follow-up without gastrectomy are compared in Table 1. The subgroup that underwent additional gastrectomy had a higher percentage of younger patients (68.8 versus 73.4 years; *p* < 0.001), positive lymphatic-vascular involvement (74.0% versus 40.3%; *p* < 0.001) and submucosal deep invasion (76.7% versus 51.9%; *p* = 0.002).

**Table 1:** Clinical characteristics of patients diagnosed as non-curative endoscopic resection with a potential risk of LNM

Factors		All (*n* = 150)	Surgery (*n* = 73)	Follow-up (*n* = 77)	*p* Value
Age		71.2	68.8	73.4	<0.001^*^
Sex	M:F	128:22	66:7	62:15	0.11
Concomitant disease		22 (14.7%)	9 (12.3%)	13 (16.9%)	0.49
Other cancer		11	5	6	
Hematologic disease		3	2	1	
Cardiovascular disease		3	2	1	
Liver cirrhosis		5	0	5	
Positive lymphatic-vascular involvement		85 (56.7%)	54 (74.0%)	31 (40.3%)	<0.001^*^
Undifferenciated type		19 (12.7%)	8 (11.0%)	11 (14.3%)	0.63
Deep submucosal invasion (≥ sm2)		96 64.0%)	56 (76.7%)	40 (51.9%)	0.0021^*^
Minute submucosal cancer (sm1 ≥ 30 mm in size)		9 (6.0%)	4 (5.5%)	5 (6.5%)	1.00
VM positive or unclear		35 (23.3%)	12 (16.4%)	23 (29.9%)	0.056
HM positive or unclear		20 (13.3%)	6 (8.2%)	14 (18.2%)	0.093

The Fisher exact test or the χ2 test was used for the analyses

*VM* vertical margin, *HM* Hiatal margin, *LNM* lymph node metastasis

Statistical significance defined as ^*^ *p* < 0.05

**Fig. 2:**
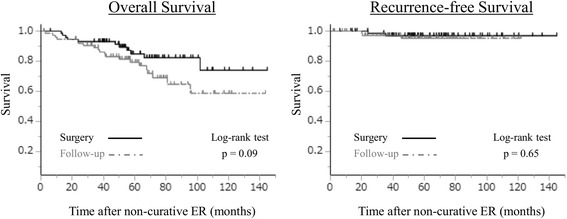
Overall and recurrence-free survival of patients with or without additional gastrectomy. ER, endoscopic resection

Overall and recurrence-free survival curves are shown in Fig. 2. Among those who underwent additional gastrectomy, the median follow-up time was 4.8 (range 0.5–11.9) years. Two of these patients (2.7%) developed distant metastasis after surgery, and died from gastric cancer. The 5-year overall and recurrence-free survivals were 85.0% and 97.0%, respectively (Fig. 2). Among those who underwent strict follow-up without gastrectomy, the median follow-up time was 4.7 (range 0.2–11.8) years. Three patients (3.9%) developed recurrence (local recurrence in two patients and LNM in one patient), and both of local recurrence patients underwent gastrectomy with lymphadenectomy and are alive without recurrence. The remaining patient who had LNM refused further treatment, and was followed up. The 5-year overall and recurrence-free survivals were 79.4% and 95.3%, respectively (Fig. 2). The 5-year overall and recurrence-free survivals of patients who underwent strict follow-up without gastrectomy were not significantly different from those who underwent gastrectomy.

The median interval between initial ER and additional gastrectomy was 88 (range 21–201) days, and there were no operation-related deaths. In additional gastrectomy specimens, 8 (11.0%) of 73 patients had LNM, which were limited in one or two peritumoral nodes. Primary tumor remained in 8 (11.0%) of 73 patients. Relationships among clinicopathological characteristics, nodal metastasis, and local residual tumor are summarized in Table 2.

**Table 2:** Results of univariate and multivariate analysis of pathological findings, remnant tumor, and lymph node metastasis

Factors	Remnant tumor		Univariate *p* Value	Multivariate *p* Value	LNM		Univariatep Value	Multivariate *p* Value
	Presence (*n* = 8)	Absence (*n* = 65)			Presence (*n* = 8)	Absence (*n* = 65)		
Lesion size (mm)	30.3	26.4	0.21	0.35	27.9	26.7	0.81	–
Lymphatic invasion	5 (62.5)	34 (52.3)	0.44	0.31	8 (100)	31 (47.7)	0.005^*^	<0.001^*^
Venous invasion	3 (37.5)	20 (30.7)	0.49	0.67	4 (50)	19 (29.2)	0.21	0.05
Undifferenciated type	2 (25)	6 (9.2)	0.21	0.32	1 (12.5)	7 (10.8)	1.00	–
Deep submucosal invasion (≥SM2)	6 (75)	50 (76.9)	0.73	–	7 (87.5)	49 (75.4)	0.67	–
VM Positive or unclear	2 (25)	10 (15.4)	0.39	0.70	1 (12.5)	11 (16.9)	1.00	–
HM Positive or unclear	3 (37.5)	3 (4.6)	0.015^*^	0.018^*^	0 (0)	6 (9.2)	1.00	–

Univariate analysis was performed by using the Fisher exact test or the χ^2^ test, and multivariate analysis was performed by using logistic regression analysis

Values in parentheses are percentages

*VM* vertical margin, *HM* hiatal margin

Statistical significance defined as ^*^ *p* < 0.05

On univariate analysis, HM was the only significant factor for local residual tumor (*p* = 0.015). For nodal metastasis, lymphatic invasion was the only significant factor (*p* = 0.005). Moreover, 8 (20.5%) of 39 patients with lymphatic invasion (ly (+)) had lymph node metastasis. On multivariate analysis, HM was the only significant factor for local residual tumor (*p* = 0.018). For LNM, only lymphatic invasion was significant (*p* < 0.001) (Table 2).

## Discussion

In this retrospective study, 707 EGC patients who were expected to satisfy the criteria for curative ER [7] underwent ER, but, in fact, 25.7% did not. Despite improvements in endoscopic examination, the endoscopic diagnosis of EGC is not always accurate, as several reports have mentioned, and is correct in only 80–90% of cases [12–14]. When patients were diagnosed as non-curative ER based on their potential risk of LNM after pathological examination following ER, additional gastrectomy with lymph node dissection was recommended [7, 15], despite the fact that the incidence of LNM of EGC is rare [9, 10]. Additional gastrectomy with lymph node dissection is necessary for EGC patients with a potential risk of LNM, but most patients without LNM have routinely undergone unnecessary surgery. The conventional gastrectomy with prophylactic lymph node dissection often has acute and chronic complications and reduces the patients’ quality of life (QOL). Thus, a specific treatment depending on the individual patient would benefit these patients by allowing them to avoid prophylactic surgery.

Patients diagnosed as non-curative ER were classified into two groups with or without a potential risk of LNM. Non-curative ER with positive HM as the only non-curative factor is generally considered to be an indication for additional local treatment (repeated-ESD or coagulation) or surgery or close observation according to the individual case [9, 16, 17]. However, it is sometimes difficult to achieve an exact diagnosis with ER specimens because of histological modifications that occur with ER [7, 18]. Several articles have reported that none of the patients diagnosed as non-curative ER due only to HM involvement developed LNM [9, 10].

The LNM rate among patients who underwent additional gastrectomy in this study was 11.0%, which was lower than the reported rate of 20% in patients with submucosal invasive cancer [5]. Some lesions with additional gastrectomy in this study had a lower risk of LNM than submucosal invasive cancer overall because they had been treated with curative intent by ER. Similar results have been reported previously, in which less than 10% of patients had LNM in surgical specimens following non-curative ER [19–21].

The 5-year overall and recurrence-free survivals among patients who underwent strict follow-up without gastrectomy were not significantly different from those in patients who underwent gastrectomy. Choi et al. [22] also reported that OS and disease-free survival did not differ significantly between patients treated with additional surgical resection and patients simply followed up after ESD in submucosa-invasive gastric cancer. However, this result should be carefully interpreted, because histological analysis demonstrated that the patients who underwent gastrectomy showed significantly higher lymphatic-vascular involvement and deeper submucosal invasion than those followed up without gastrectomy (Table 1), indicating that the patients with these unfavorable histological findings were more frequently selected for additional gastrectomy. Kawata et al. [20] and Suzuki et al. [21] reported that there was a significant difference in OS between additional surgery and follow-up groups, although disease-specific survival did not differ significantly between the two groups. In patient clinical backgrounds, the follow-up group was significantly older than the additional surgery group, and several patients died of causes other than gastric cancer. Therefore, advanced age or concomitant disease may have contributed to the poor prognosis of the follow-up group. In the current study, all patients in the follow-up group died of causes other than gastric cancer during the study period. This result indicates that strict follow-up instead of additional surgery may be an acceptable management option for certain patients with diagnoses with non-curative ER. Moreover, risk stratification associated with LN or distant metastasis and gastric cancer related death of non-curative ER patients is required for an appropriate treatment strategy.

In this study, 8 of 39 patients (20.5%) with positive lymphatic invasion and 4 of 23 (17.4%) with positive venous invasion had metastasis in regional LNs. Only lymphatic invasion was an independently significant factor of LNM (*p* < 0.001), and there was no LNM in lesions without lymphatic invasion. This result indicates that additional gastrectomy with lymphadenectomy should be performed for lesions with lymphatic invasion. Some similar studies [20, 21] reported that lymphatic invasion was an independent risk factor for LNM in non-curative ER patients. Furthermore, all metastatic nodes were located in the perigastric area close to the primary tumors. This suggests that minimizing the lymphadenectomy and reducing the extent of gastrectomy as additional surgery following non-curative ER may be acceptable. Some articles have reported that function-preserving gastrectomy, such as pylorus-preserving gastrectomy and proximal gastrectomy, improved postoperative QOL, including postoperative symptoms, weight gain, and food intake volume [23–25]. However, inadequate treatment may yield remnant metastatic LNs. In recent years, the validity of sentinel node navigation surgery for EGC was reported by some studies [26, 27]. Though it is unclear whether the sentinel hypothesis is suitable for EGC after ER, function-preserving surgery may be used for patients who have been diagnosed as non-curative ER in the future [28].

The limitations of this study are its retrospective, single-center design, and the differences in the clinicopathological background characteristics of the two groups. A prospective randomized clinical trial (RCT) is needed to establish more appropriate treatment strategies for non-curative ER patients, although it may be difficult to conduct a prospective RCT for ethical reasons.

## Conclusion

Additional gastrectomy with lymphadenectomy is strongly recommended for patients with lymphatic invasion among patients diagnosed as non-curative ER due to their potential risk of LNM, and minimizing the extent of lymphadenectomy may be allowed for these patients. However, a RCT is required to establish more appropriate treatment strategies for these patients.

## Abbreviations

EGC: Early gastric cancer
EMR: Endoscopic mucosal resection
ER: Endoscopic resection
ESD: Endoscopic submucosal dissection
HM: Hiatal margin
LNM: Lymph node metastasis
OS: Overall survival
QOL: Quality of life
RCT: Randomized clinical trial
VM: Vertical margin
